# Primary Cardiac Sarcoma: Angiosarcoma Compressing the Right Coronary Artery

**DOI:** 10.7759/cureus.38360

**Published:** 2023-04-30

**Authors:** Mohamad Mubder, Issa Pour-Ghaz, Omar Al-Taweel, Buthainah Alhwarat, Addison Bond, Ahsan H Choudhury, Osama Mahmoud, Deya Alkhatib

**Affiliations:** 1 Internal Medicine, Kirk Kerkorian School of Medicine at University of Nevada, Las Vegas, Las Vegas, USA; 2 Cardiology, The University of Tennessee Health Science Center, Memphis, USA; 3 Cardiology, Kirk Kerkorian School of Medicine at University of Nevada, Las Vegas, Las Vegas, USA; 4 Faculty of Medicine, The Hashemite University, Zarqa, JOR; 5 College of Medicine, The University of Tennessee Health Science Center, Memphis, USA; 6 Cardiology, University Medical Center, Las Vegas, USA; 7 Cardiology, University of Nevada, Reno, USA; 8 Cardiovascular Medicine, The University of Tennessee Health Science Center, Memphis, USA

**Keywords:** coronary artery disease, soft tissue sarcoma, right atrial myxoma, sarcoma soft tissue, cardiac mass tumor, cardiac angiosarcoma

## Abstract

Cardiac tumors are uncommon and can be classified as either primary benign, primary malignant, or metastatic. Cardiac tumors have a wide range of presentations, which can lead to delays in diagnosis and treatment. Primary cardiac tumors can also affect nearby structures, and there have been a few reported cases of coronary artery involvement with various underlying causes. In this case report, we describe a patient with a primary cardiac sarcoma (angiosarcoma) that had spread to other parts of the body and caused occlusion of the right coronary artery.

## Introduction

Cardiac tumors are rare findings for both primary and metastatic types, with the most common being metastatic tumors. Primary cardiac tumors only occurred at an incidence of up to 0.03% in an autopsy series, with metastatic cancers to the heart being roughly 30-50 times more common [1]. Cancers with the highest frequency of metastasis to the heart include lung, breast, melanoma, lymphomas, and renal cell cancer, with the lung being the most common.

Primary cardiac tumors are divided into benign (>90%) and malignant (<10%). The most common malignant primary cardiac tumors are cardiac sarcomas, including angiosarcomas, leiomyosarcoma, rhabdomyosarcoma, and fibrosarcoma. Due to the difficulty of obtaining a biopsy and relying on imaging characteristics to make a presumptive diagnosis, diagnosis and management can be challenging [2].

This case was presented as a poster at the local American College of Cardiology (ACC) Chapter meeting (ACC Tennessee Chapter) in November 2022.

## Case presentation

**Figure 1:**
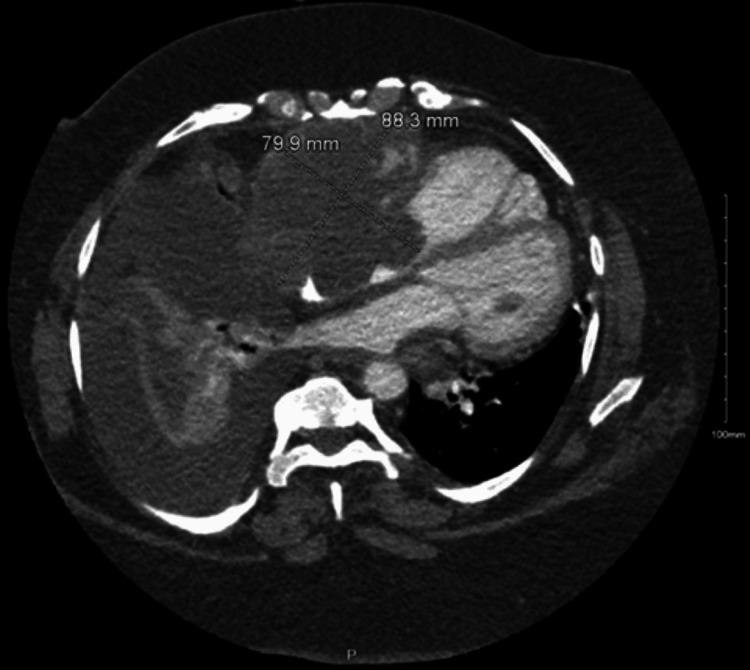
Chest CT showing a large lobulated mass in the right atrium measuring 8.8 cm x 8 cm.

The patient was a 64-year-old woman with a past medical history of diabetes mellitus, ocular stroke, hyperlipidemia, uterine cancer, and hypothyroidism, who presented to the hospital due to shortness of breath. Chest computed tomography (CT) was significant for scattered, ill-defined nodules with a large right pleural effusion throughout both lungs. Additionally, chest CT showed a large lobulated mass originating in the right atrium (measuring approximately 8.8 x 8.0 cm) and a 2.0 x 1.5 cm nodule in the right pericardium (Figure 1).

**Figure 2:**
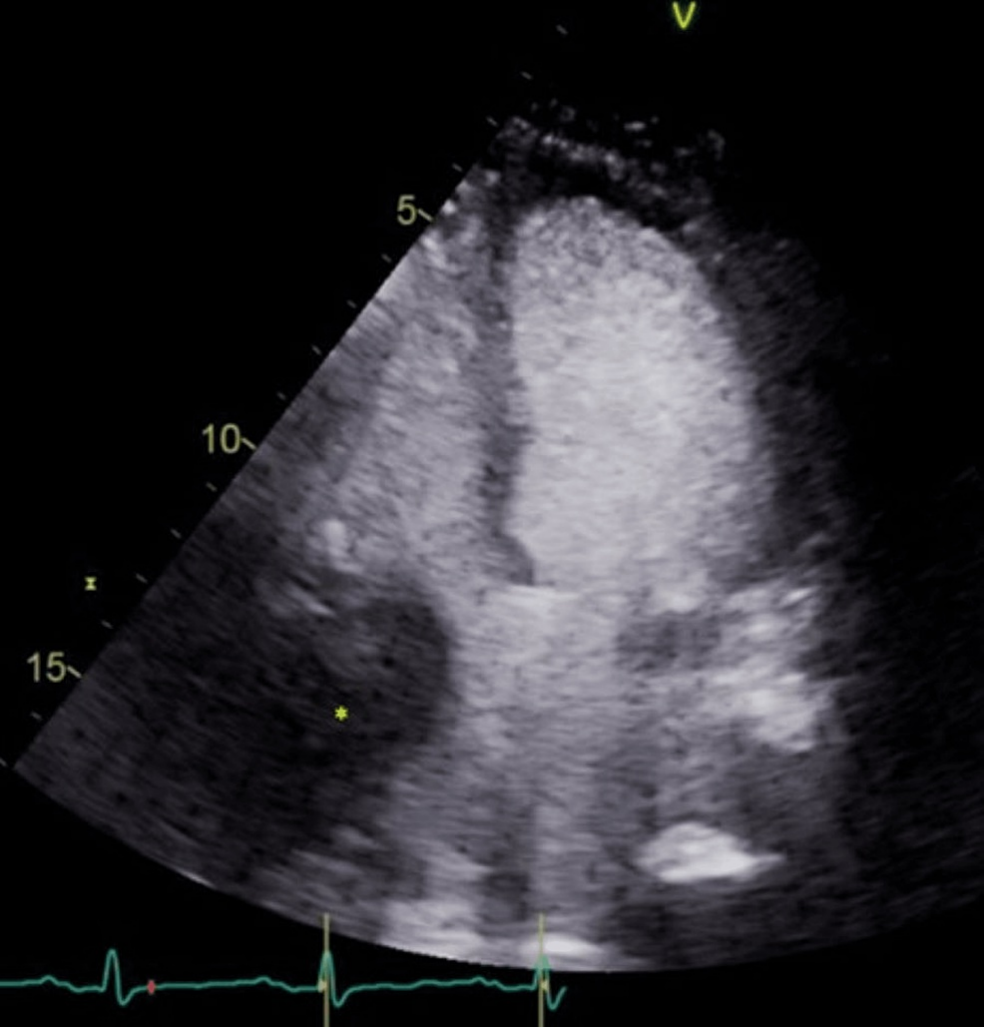
Transthoracic echocardiogram with the ultrasound-enhancing agent showing the right atrial mass (*).

A transthoracic echocardiography (TTE) was significant for a moderately dilated right atrium, a large lobulated mass in the right atrium, and normal biventricular systolic function (Figure 2).

**Figure 3:**
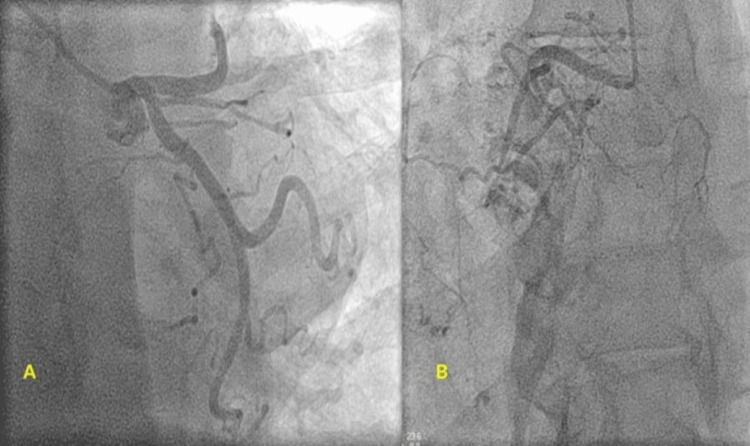
Coronary angiogram showing normal left coronary artery system (A) and 100% occluded mid-right coronary artery (B).

Thoracentesis and video-assisted thoracoscopic biopsy of the ill-defined mass was performed. Biopsy results were consistent with high-grade angiosarcoma of the right heart (primary cardiac angiosarcoma) with metastasis to the lungs, pleural space, liver, and brain. Preoperative cardiac evaluation for surgery was then pursued. The patient underwent cardiac catheterization with coronary angiography, which showed a normal left coronary system and a 100% occlusion in the mid-segment of the right coronary artery (RCA) consistent with tumor compression (Figure 3).

She was started on radiotherapy for the large right atrial mass with plans to initiate systemic chemotherapy. However, after completing the radiotherapy session, her overall status deteriorated with respiratory failure and encephalopathy. After a thorough discussion with the family and a multidisciplinary team (including the palliative care team), the family opted to pursue comfort measures and hospice care. The patient passed away a few days after.

## Discussion

Cardiac tumors are a heterogeneous group of disorders and include primary benign and malignant tumors, as well as metastatic disease [2]. Presentation could range from incidental findings to general symptoms, like a low-grade fever, weight loss, night sweats, and fatigue, to more significant symptoms, like obstructive symptoms, as seen in superior vena cava syndrome, arrhythmia, and embolism [3,4].

A more significant tumor burden is observed with cardiac sarcomas involving the right side of the heart. Given the rare incidence of cardiac tumors, the investigation should begin by differentiating cardiac tumors from other causes [2,3]. Cardiac tumors are diagnosed using multimodality non-invasive imaging techniques. Of the available cardiac imaging modalities, TTE is the first diagnostic test used. Differential diagnosis of a cardiac mass on TTE includes thrombus, vegetation, metastatic neoplasm, primary cardiac tumor, and artifact (unusual echocardiographic plane or normal variant). Other imaging modalities include cardiac CT (CCT), cardiac magnetic resonance (CMR), and positron emission tomography (PET). They can help to narrow down the differential diagnosis, characterize the tissue mass, and detect primary metastatic locations in case of metastatic cardiac tumors [2,5].

Approximately 90% of primary cardiac tumors are benign. Atrial myxoma is the most common primary cardiac tumor, comprising up to 50% of all primary cardiac tumors. Approximately 10% of primary cardiac tumors are malignant [3,6-8]. Primary cardiac sarcoma represents only 1% of all soft tissue sarcomas. Patients with cardiac sarcoma can present at a wide range of ages (the mean age of presentation of 40 years) [9]. Angiosarcomas and unclassified sarcomas represent approximately 75% of all cardiac sarcomas, with angiosarcomas being the most common type. Other cardiac sarcomas include rhabdomyosarcoma (the most common type of cardiac sarcoma in children), leiomyosarcoma, osteosarcoma, fibrosarcoma, and liposarcoma. Some cardiac tumors have the tendency to affect a particular side or chamber of the heart. Angiosarcomas are typically found in the right side of the heart (right atrium and atrioventricular groove). In contrast, unclassified sarcomas and osteosarcomas are usually found on the left side of the heart [10].

Cardiac sarcomas, like other cardiac tumors, cause symptoms through three mechanisms: mechanical obstruction, arrhythmias, and embolization. Patients can present with shortness of breath (the most common presenting symptom), chest pain, syncope (due to obstruction of intracardiac flow), palpitations, general symptoms (fever, night sweat, and weight loss), distal embolic events, and sudden cardiac death. At the time of presentation, about one-third of cardiac sarcomas have distant metastasis, most commonly in the lungs [11,12].

Involvement of the coronary arteries has rarely been reported in the literature. Different mechanisms of causing obstructive coronary artery disease were reported, including direct invasion of coronaries with metastatic lesions or mechanical obstruction at the ostium causing recurrent angina [13,14].

Due to the exceedingly rare incidence of cardiac sarcomas, managing patients with cardiac sarcomas depends on individual physicians experienced with this disease. As a result, treating such patients require a multidisciplinary cardiac tumor team that includes cardiac imaging experts, cardio-oncologists, and specialized cardiac surgeons [15]. Cardiac sarcomas, especially right atrial sarcomas, require immediate surgical attention to mitigate the risk of secondary complications [2,8]. The ultimate goal of therapy is a complete surgical resection of the tumor, which can be followed by adjuvant chemotherapy to improve survival. As opposed to other sarcomas, cardiac sarcomas have a very poor prognosis, especially in the presence of distant metastasis. If surgery cannot be performed, the one-year survival of cardiac sarcoma is less than 10% [7,15].

Total surgical resection continues to be the treatment option of choice. The survival rate correlates directly with the extent of resection based on the retrospective analysis of the French sarcoma group. However, in many cases, the clinical presentation does not allow radical surgical excision, which limits the impact on prognosis, which remains extremely poor [16,17].

Heart transplant has recently been introduced as an emerging strategy for patients with isolated inoperable cardiac involvement. Other treatment options include chemotherapy and radiation therapy, which historically represented a palliative approach for patients who were not eligible for surgery. Nevertheless, the latest technological developments in the field of radiation oncology may expand the current treatment options, leading to clinical outcome improvement [17].

## Conclusions

Primary malignant tumors that originate in the heart are known to be highly aggressive and often lead to poor patient outcomes, even with the currently available treatment options. The case we present here is an unusual example of a primary cardiac sarcoma (angiosarcoma) that had spread to other parts of the body and was compressing the RCA from the outside. To diagnose and locate the tumor, we relied on a combination of tests, including TTE and advanced cardiac imaging techniques such as CCT, CMR, and PET. Whenever possible, complete surgical removal of the tumor is the preferred approach, while chemotherapy and radiation therapy may be considered on a case-by-case basis.
